# P-1344. Clinical outcomes of patients with NDM-producing Enterobacterales bloodstream infections in a healthcare system in New York

**DOI:** 10.1093/ofid/ofaf695.1532

**Published:** 2026-01-11

**Authors:** Santosh Dahal, Pranita Tamma, Marcia Epstein, Rubab Sohail, Meredith Akerman, Pranisha Gautam-Goyal, Aya Haghamad, Vincent Streva, Miriam A Smith, Bruce Hirsch, Joanna DeAngelis, Nardine Karam, Samantha H Cham, Tina Zheng, Sumeet Jain, Patricia Saunders-Hao

**Affiliations:** Northwell - Long Island Jewish Medical Center, New Hyde Park, New York; Johns Hopkins University School of Medicine, Baltimore, Maryland; Donald and Barbara Zucker School of Medicine at Hofstra/Northwell, Manhasset, New York; Northwell Health, New Hyde Park, New York; Northwell Health, New Hyde Park, New York; Zucker School of Medicine at Hofstra/Northwell, Manhasset, New York; Northwell, Lake Success, New York; Northwell Health Laboratories, Queens, NY; Donald and Barbara Zucker School of Medicine at Hofstra/Northwell, Manhasset, New York; Hoftsa Northwell School of Medicine, Manhasset, NY; Staten Island University Hospital, Staten Island, New York; Arnold & Marie Schwartz College of Pharmacy and Health Sciences, Long Island University, Staten Island, New York; Maimonides Medical Center, Brooklyn, New York; Maimonides Health, Brooklyn, New York; North Shore University Hospital, Westbury, NY; North Shore University Hospital, Westbury, NY

## Abstract

**Background:**

New Delhi Metallo-beta-lactamase (NDM)-producing Enterobacterales infections pose a significant health threat given limited therapeutic options. A rise in cases within our health system prompted us to compare clinical outcomes of patients with NDM-producing bacteremia treated with ceftazidime/avibactam plus aztreonam (CZA-ATM) and/or cefiderocol (FDC).

**Figure:**
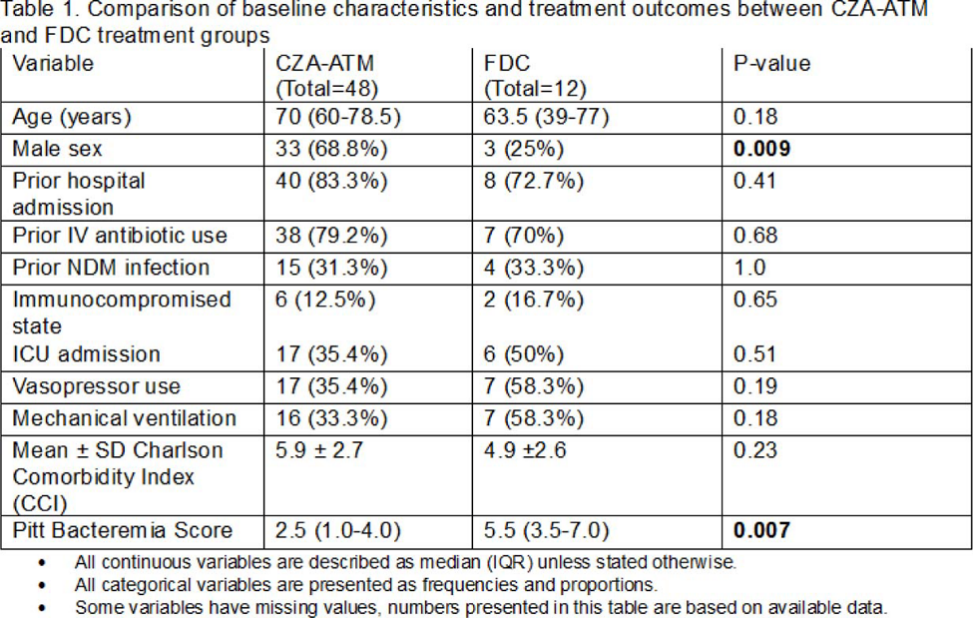

**Figure:**
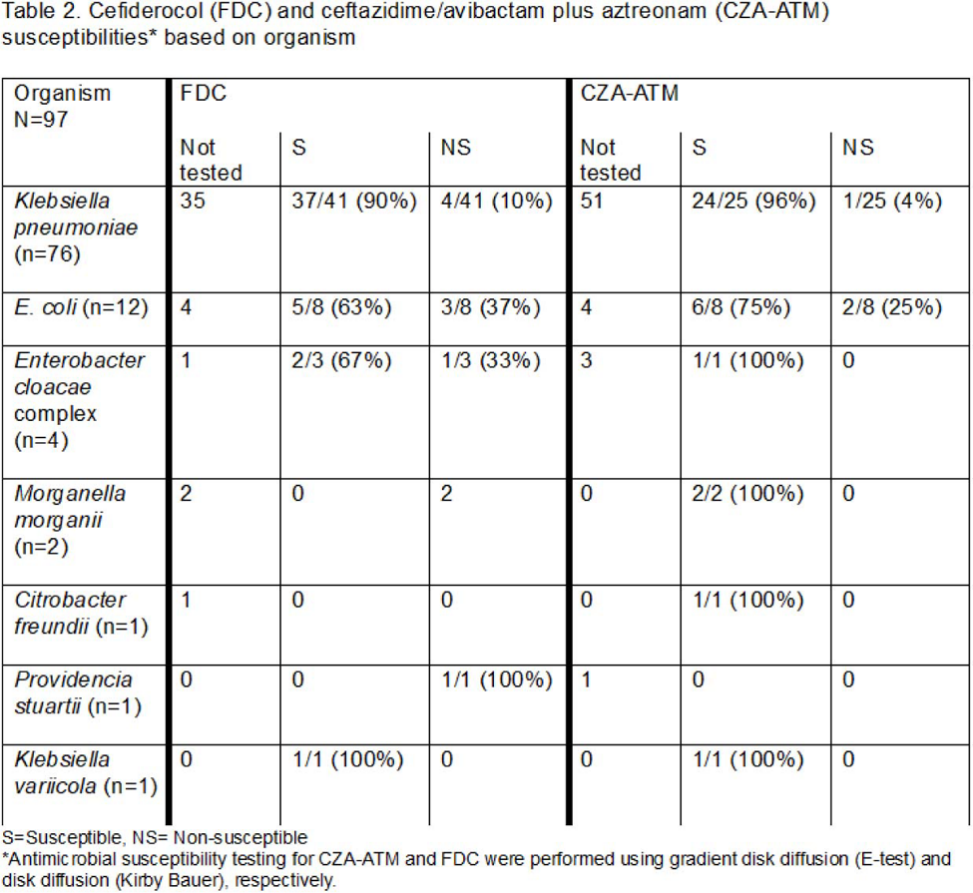

**Methods:**

Patients identified to have blood cultures with an NDM-producing organism (detection of *bla*_NDM_ by PCR) from 2022 to 2024 were analyzed retrospectively. Only patients who completed therapy with an active agent (CZA-ATM or FDC) were included in the analysis. Baseline characteristics were compared using Fisher’s Exact test and Wilcoxon rank-sum test, as appropriate. Logistic regression, adjusting for baseline variables with p-values of < 0.10, was used to determine the adjusted odds ratio of mortality at 30 days.

**Results:**

A total of 97 infections were identified. The most common organisms were *Klebsiella pneumoniae* (78%, n=76) and *E. coli* (12%, n=12). Eight percent (3/38) of isolates were non- susceptible to CZA-ATM and 17% (9/54) were non-susceptible to FDC. Seventeen (17%) patients died within 48 hours from culture. Of the 60 patients who received targeted therapy without a regimen change, 48 (80%) patients received CZA-ATM and 12 (20%) received FDC. Median (IQR) age of patients was 70 (60-78.5) years, 23 (34%) were admitted to an ICU within 24 hours of culture collection, and 8 (13%) were immunocompromised. Baseline characteristics in the two groups were similar, except for male sex and Pitt bacteremia score. A total of 22 (37%) patients died by 30 days (17 (35%) in CZA-ATM group and 5 (42%) in FDC group) but this was not significantly different (OR=1.30; 95% CI: 0.36-4.74; p=0.688). Days to bacteremia clearance was significantly higher in the FDC group (p=0.02). Recurrent infection at 90 days occurred in 13 (27%) and 3 (25%) in the CZA-ATM and FDC groups, respectively (p=1.000).

**Conclusion:**

Infections with NDM-producing organisms are associated with high mortality. FDC resistance may be a concern for its empiric use. Although a larger cohort is required, our data suggest that mortality and likelihood of recurrent infection are similar whether CZA-ATM or FDC is used for treatment of NDM bacteremias.

**Disclosures:**

All Authors: No reported disclosures

